# Serum Mature and Precursor Brain-Derived Neurotrophic Factors and Their Association with Neurocognitive Function in ART-Naïve Adults Living with HIV in Sub-Saharan Africa

**DOI:** 10.1007/s12035-024-04599-2

**Published:** 2024-11-16

**Authors:** Henry U. Michael, Antony M. Rapulana, Theresa Smit, Njabulo Xulu, Sivapragashini Danaviah, Suvira Ramlall, Frasia Oosthuizen

**Affiliations:** 1 Discipline of Pharmaceutical Sciences, School of Health Science, University of KwaZulu-Natal, Durban, South Africa; 2 Africa Health Research Institute, Durban, South Africa; 3 Faculty of Applied Science, Eduvos, Midrand, South Africa; 4 Department of Psychiatry, University of KwaZulu-Natal, Durban, South Africa; 5 School of Laboratory Medicine and Medical Science, University of KwaZulu-Natal, Durban, South Africa; 6 UCL Centre for Clinical for Clinical Microbiology, Division of Infection & Immunity, University College London, London, England; 7 Division of Clinical and Translational Research, McGill University, Montreal, QC Canada; 8 Centre for Outcomes Research & Evaluation, Research Institute of McGill University Health Centre (RI-MUHC), Montreal, QC Canada

**Keywords:** Brain-derived neurotrophic factor, HIV, Cognition, Sub-Saharan Africa

## Abstract

This study investigates the association between serum mature brain-derived neurotrophic factor (mBDNF), its precursor proBDNF, and neurocognitive function in ART-naïve adults with HIV in sub-Saharan Africa, exploring the distinct roles of these neurotrophic factors in cognitive health. This cross-sectional analysis utilized stored baseline serum samples and neuropsychological test data from participants in the AIDS Clinical Trials Group (ACTG) A5199 study in the Johannesburg and Harare sites. Serum concentrations of mBDNF and proBDNF were quantified using ELISA. Neurocognitive function was assessed via standardized tests, with results adjusted for site-specific demographics. Linear and quantile regression models examined the relationship of mBDNF and proBDNF with a composite cognitive score (NPZ-6), and structural equation modeling (SEM) explored their association with individual cognitive test outcomes. The analysis involved 157 ART-naïve adults with HIV. Increased serum mBDNF levels showed a significant positive association with cognitive performance (*β* = 1.30, *p* = 0.02), while elevated proBDNF levels were linked to poorer outcomes, particularly affecting fine motor skills and speed (*β* =  − 0.29 to − 0.38, *p* ≤ 0.01). Quantile regression analysis highlighted mBDNF’s stronger positive impact at higher cognitive performance percentiles (*β* = 1.04 (0.01, 2.06) at the 75th percentile), while proBDNF showed significant negative association at the 75th percentile (*β* =  − 0.26 (− 0.47, − 0.06)). The study highlights the positive association of mature BDNF and the negative association of proBDNF with cognitive function in HIV. These findings emphasize the need for longitudinal research to understand the temporal dynamics of neurotrophic factors during ART initiation and their potential as targets for neurocognitive interventions in HIV.

**Supplementary Information:**

The online version contains supplementary material available at 10.1007/s12035-024-04599-2.

## Introduction

As of 2023, UNAIDS reports that 39 million people globally are living with HIV, with two-thirds of these cases in sub-Saharan Africa [1]. In this region, over 82% of people with HIV are on antiretroviral therapy (ART), reducing AIDS-related deaths. However, asymptomatic and mild HIV-associated neurocognitive impairment (NCI) remains prevalent. HIV-associated NCI affects daily activities, work, medication adherence, and overall quality of life for people with HIV [2].

One reason for ongoing NCI, even with viral suppression, is the early impact of HIV on the brain before starting ART [3]. This is particularly relevant in sub-Saharan Africa, where late healthcare access and ART initiation lead to prolonged advanced immunosuppression, adversely affecting brain reserve and cognition [4, 5].

The neuropathogenesis of HIV-associated neurocognitive impairment (NCI) is driven by complex mechanisms, including the diminished synthesis of neurotrophins, which disrupts neuroplasticity—the CNS’s inherent ability for structural and functional reorganization in response to environmental stimuli and neural challenges [6, 7]. Central to this process is brain-derived neurotrophic factor (BDNF), crucial for synaptogenesis, neuronal differentiation, and survival, hence its potential as a molecular biomarker for neurological disorders with impaired cognitive and motor function such as Alzheimer’s, Parkinson’s, and Huntington’s diseases, and HIV-associated NCI [8–11]. Viral proteins, including gp120 and Tat, have been implicated in the dysregulation of brain-derived neurotrophic factor (BDNF) maturation, inhibiting the conversion of its precursor form, proBDNF, into mature BDNF (mBDNF) [7]. Human mBDNF is synthesized as proBDNF, which undergoes cleavage by intracellular and extracellular enzymes to convert into its mature form [12]. The functional distinction between proBDNF and mBDNF is significant, with proBDNF signaling through the p75 neurotrophin receptor (p75NTR), contributing to processes like synaptic pruning and apoptosis, while mBDNF binds to the tropomyosin receptor kinase B (TrkB) receptor, promoting neuronal survival and synaptic plasticity [13, 14]. This dichotomy underscores the complex interplay between viral pathogenicity and neurotrophic signaling, with implications for understanding the neuropathogenesis of viral infections.

The overlap between immune response and BDNF concentrations is particularly evident in HIV-induced neuroinflammation. Neuroinflammatory cytokines like TNF-α and IL-1β, produced during HIV infection, inhibit BDNF expression and disrupt its transport within neurons [15]. Additionally, the kynurenine pathway, activated by immune and metabolic dysfunctions linked to HIV, directly influences BDNF signaling [15]. Pro-inflammatory processes can significantly reduce BDNF levels, impairing neuroplasticity and contributing to cognitive decline [15–17]. This interaction underscores the critical role of immune response in modulating BDNF signaling, which is highly relevant to HIV-related neurocognitive impairment.

Several clinical investigations have documented an association between diminished levels of cerebrospinal fluid [18], plasma [19], and serum whole BDNF [20] with HIV-related cognitive impairment, as evidenced by decreased performance in neurocognitive assessments. Despite these findings, there remains a conspicuous absence of studies elucidating the differential impact of precursor BDNF (proBDNF) and mature BDNF on neurocognitive function in this population. The period preceding ART initiation, characterized by immunosuppression, presents a critical window to explore the distinct roles of proBDNF and mature BDNF in the cognitive process of persons with HIV. An enhanced understanding of the equilibrium between proBDNF and mature BDNF in such individuals could be pivotal in the development of biomarkers for the early detection of neurocognitive decline, thereby augmenting patient care in the initial phase of HIV management. Furthermore, this knowledge could inform the innovation of therapeutic strategies specifically targeting these neurotrophins, with the objective of preserving or ameliorating cognitive functionality in the context of HIV.

The primary objective of this investigation is to estimate the associations between mature BDNF, its precursor proBDNF, and the ratio of mature BDNF to proBDNF, with cognitive function in ART-naïve adults diagnosed with HIV in sub-Saharan Africa. Additionally, the study employs quantile regression to examine the influence of mature BDNF and proBDNF across varying levels of cognitive performance. Furthermore, structural equation modeling (SEM) is utilized to investigate in detail the associations of mature BDNF and proBDNF with specific neurocognitive test outcomes. We hypothesize that increased levels of mature BDNF are positively associated with cognitive function, independent of confounding factors, whereas proBDNF is expected to exhibit an inverse relationship with cognitive function.

## Methods

### Participants and Study Design

This study was a secondary cross-sectional analysis of stored baseline serum samples and neuropsychological test data obtained from a subset of participants who were enrolled in the Johannesburg, South Africa, and Harare, Zimbabwe, sites of the AIDS Clinical Trials Group (ACTG) A5199 study from 2006 to 2010. The A5199 study was a neurological sub-study of the PEARLS study (A5175), which was a multinational randomized trial across resource-limited settings focused on treatment strategies for people living with HIV starting their first ART regimen. The methodologies and outcomes of A5199 (ClinicalTrials.gov NCT00096824) and A5175 (ClinicalTrials.gov NCT00084136) have been detailed in prior publications [21, 22]. Key inclusion criteria for the A5199 study were an age > 18 years, a documented HIV-1 infection, a CD4 count < 300 cells/mm^3^, a Karnofsky performance score > 70, limited prior ART exposure (less than 7 days), absence of significant psychiatric pathologies, and no active substance abuse or dependence at study entry determined by site clinician.

For this analysis, participants were chosen through criterion-based purposive sampling: enrollment at the Johannesburg or Harare sites of the A5199 study, availability of neuropsychological test results, presence of pre-stored serum samples, and consent for specimen utilization. Original participant consent was acquired during the parent study, adhering to ethical protocols. This analysis received approval from the Human Research Ethics Board of each participating site and the University of KwaZulu-Natal Biomedical Research Ethics Committee.

### Measurement

#### Laboratory Measurements of mBDNF and ProBDNF

Serum concentrations of mature brain-derived neurotrophic factor (mBDNF) and its precursor proBDNF were quantitatively assessed using commercially available enzyme-linked immunosorbent assay (ELISA) kits (Biosensis Pty Ltd., Thebarton, SA, Australia). The serum specimens, previously stored at − 70 °C, were processed in accordance with the manufacturer’s protocol. To minimize assay variance, serum levels of proBDNF and mBDNF from each subject were measured on the same plate to reduce the potential variation between plates/kits. All samples were tested in duplicate, and the two tests were averaged. The intra-assay coefficients of variation for the mBDNF and proBDNF were 2.7% and 2.2%, respectively. Analysts performing the assays were blinded to the clinical characteristics and neuropsychological test results of participants. Serum mBDNF and proBDNF were log-transformed for analysis due to right skewness.

#### Neuropsychological Assessment

Participants at both research sites underwent a battery of standardized neurocognitive assessments designed to evaluate a range of cognitive and motor functions. Fine motor dexterity and speed were assessed using the Grooved Pegboard Test for both dominant and non-dominant hands, as well as the Finger Tapping test. Gross motor skills and gait velocity were measured using the Timed Gait test, and verbal fluency was assessed via the semantic verbal fluency test [23–25]. These specific neurocognitive tests were selected to ensure brevity and practicability across multicenter trials in various resource-constrained settings, while also minimizing the influence of linguistic and cultural biases [26]. All test scores were standardized into z-scores adjusted for site, age (above and below 35 years), sex (male/female), and education (above and below 10 years) using normative data from the International Neurocognitive Normative Study (INNS ACTG A5271), which drew participants from HIV testing centers and local clinics to reflect the at-risk population [27]. Z-scores of Grooved Pegboard and Timed Gait tests were computed to let higher scores reflect better performance. The primary outcome was the NPZ-6 composite score, representing overall neurocognitive function, calculated as the mean of individual test z-scores; lower scores denoted reduced cognitive ability. As secondary outcomes, z-scores for each test were analyzed independently, with scores above the mean indicating better than average cognitive performance.

#### Covariates

Covariates were selected a priori based on prior empirical evidence highlighting their confounding potential in the relationship between serum brain-derived neurotrophic factor (BDNF) levels and cognitive performance. These covariates included age (quantified in years) at the initial study visit, years of educational attainment, biological sex, body mass index (BMI, calculated as kg/m^2^), the CD4 to CD8 cell count ratio at study entry, log-transformed plasma HIV RNA levels, and the specific study site from which the data were collected.

#### Statistical Analysis

Baseline characteristics were detailed using descriptive statistics. For continuous variables, differences between sites were evaluated using the *t*-test or Mann–Whitney *U* test, depending on the data distribution. Categorical variables were compared using the chi-square test or Fisher’s exact test, as appropriate.

Three linear regression models were developed. Model 1 evaluated the association of NPZ-6 (composite measure of cognitive function) with log-transformed mBDNF levels, adjusting for confounding factors including age, sex, education, study site, CD4:CD8 ratio, HIV viral load, and BMI. Model 2 assessed the relationship between NPZ-6 and log-transformed proBDNF levels, and model 3 examined the association with the log-transformed mature BDNF to proBDNF ratio. Forest plots illustrated these associations visually. Each model’s assumptions were rigorously tested, including linearity (assessed via scatterplots of residuals against fitted values), independence (Durbin-Watson test), homoscedasticity (Breusch-Pagan test and residuals vs. fitted values plot), and normality of residuals (histogram, QQ-plot, and Shapiro–Wilk test). Additionally, multicollinearity was assessed using variance inflation factors (VIFs). Outliers were identified using the standard deviation and interquartile range methods, complemented by Cook’s distance to detect influential observations. We conducted sensitivity analyses by excluding these outliers.

Quantile regression analyses at the 25%, 50% (median), and 75% quantiles were conducted for both mBDNF and proBDNF. This approach allowed for an understanding of the relationship across the distribution spectrum of cognitive function.

In addition, we employed structural equation modeling (SEM) to further examine the complex interrelationships between neurotrophic factors, demographic variables, and individual cognitive test scores. The SEM was estimated using the weighted least squares mean and variance adjusted (WLSMV) method to accommodate the non-normal distribution of some variables. Model fit was evaluated using various fit indices including Comparative Fit Index (CFI ≥ 0.95), Tucker-Lewis Index (TLI ≥ 0.95), root mean square error of approximation (RMSEA ≤ 0.05), and standardized root mean square residual (SRMR ≤ 0.08). To improve model fit, modification indices were inspected, suggesting potential paths for inclusion in a revised model.

All tests were two-sided, and the level of statistical significance was set at *p* < 0.05. All statistical analysis was conducted using R software version 4.3.1 (R Core Team, 2023, Vienna, Austria).

## Results

### Participant Characteristics

Table 1 details the demographic, clinical, and neuropsychological profile of 157 ART-naïve adults with HIV from two sub-Saharan African sites. The cohort’s median age was 35.9 years, with females constituting 66.2% of participants. Educational levels were similar across sites, averaging 11 years. Clinically, the median CD4 count was 191 cells/mm^3^, with significant differences observed between sites (*p* = 0.005). Variations were also noted in BMI and CD4:CD8 ratios. Log-transformed median mBDNF, proBDNF levels, and mBDNF:proBDNF ratios varied between sites, as did neuropsychological test scores, with no significant difference in the composite NPZ-6 score across sites (*p* = 0.14).

**Table 1:** Demographic, clinical, and neurocognitive characteristics of study participants stratified by study site

Characteristics	All participants (*N* = 157)	Johannesburg site (*N* = 77)	Harare (*N* = 80)	*p*-value
Demographics				
Age, mean (SD)	35.9 (7.59)	34.7 (7.66)	37.0 (7.40)	0.06
Sex, *n* (%)				
Female	104 (66.2%)	55 (71.4%)	49 (61.3%)	0.24
Male	53 (33.8%)	22 (28.6%)	31 (38.8%)	
Years of education, median [IQR]	11 [9, 12]	11 [10, 12]	11 [9, 12]	0.45
BMI, median [IQR]	22.7 [20.87, 26.40]	24.5 [21.95, 28.30]	21.6 [20.36, 24.06]	< 0.001
HIV-related variables, median [IQR]				
CD4 (cells/mm^3^)	191 [130, 227]	200 [158, 240]	172 [99, 217]	0.005
CD8 (cells/mm^3^)	890 [580, 1196]	795 [564, 1127]	890 [580, 1196]	0.33
CD4:CD8 ratio	0.22 [0.13, 0.29]	0.23 [0.17, 0.33]	0.19 [0.09, 0.24]	< 0.001
Log plasma HIV RNA (copies/ml)	5.03 [4.51, 5.50]	4.97 [4.50, 5.48]	5.14 [4.55, 5.54]	0.29
BDNF variables, median [IQR]				
Log10 mBDNF (pg/ml)	1.95 [1.86, 2.02]	1.90 [1.83, 2.00]	1.97 [1.90, 2.03]	0.01
Log10ProBDNF (pg/ml)	1.61 [1.15, 2.17]	1.91 [1.29, 2.33]	1.44 [1.04, 1.93]	0.01
Log10 BDNF: Log10 ProBDNF ratio	1.12 [0.84, 1.66]	1 [0.81, 1.38]	1.29 [0,89, 1.81]	0.02
Neuropsychological test z-scores, median [IQR]				
Grooved Pegboard (dominant hand)	− 0.2 [− 0.9, 0.35]	− 0.01 [− 0.47, 0.45]	− 0.41 [− 1.39, 0.17]	0.002
Grooved Pegboard (non-dominant hand)	− 0.12 [− 0.85, 0.29]	0 [− 0.53, 0.31]	− 0.5 [− 1.58, 0.21]	< 0.001
Finger Tapping (dominant hand)	0.72 [− 0.20, 1.78]	0.34 [− 0.64, 1.06]	1.50 [0.30, 2.16]	< 0.001
Finger Tapping (non-dominant hand)	0.55 [0.49, 1.75]	0.09 [− 0.81, 0.98]	1.32 [0.09, 2.37]	< 0.001
Timed Gait	− 0.61 [− 1.53, 0.15]	− 1.10 [− 1.94, 0.34]	− 0.26 [− 1.22, 0.66]	0.002
Semantic verbal fluency	0.27 [− 0.71, 2.07]	2.07 [0.85, 3.69]	− 0.79 [− 1.64, − 0.08]	< 0.001
Composite score (NPZ-6)	0.18 [− 0.39, 0.70]	0.24 [− 0.18, 0.70]	0.14 [− 0.75, 0.69]	0.14

Cognitive scores were standardized based on the norms from the ACTG A5271 study [27]

Abbreviations: *BDNF* brain-derived neurotrophic factor, *mBDNF* mature brain-derived neurotrophic factor, *BMI* body mass index

### Serum mBDNF and Cognitive Function

**Fig. 1:**
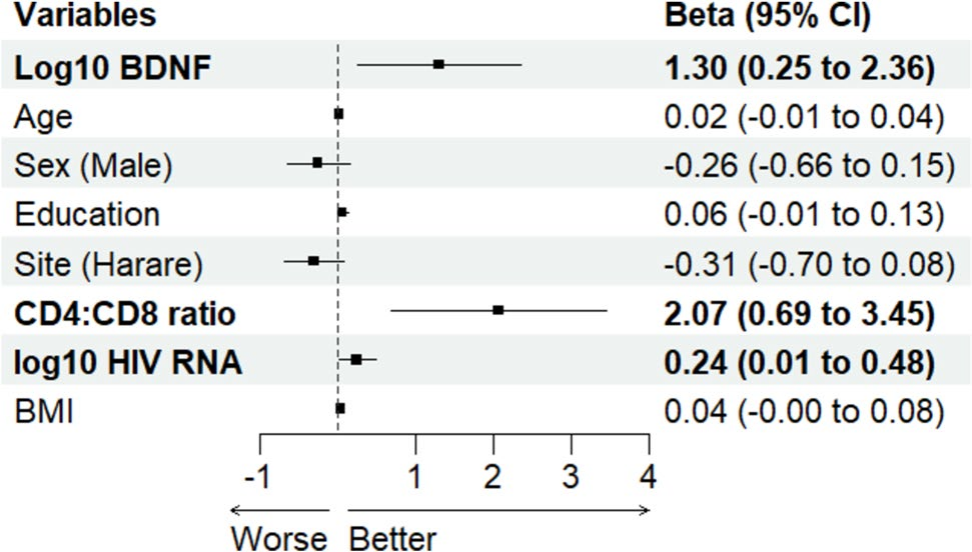
Forest plot showing the association between log-transformed mature BDNF and composite cognitive score (NPZ-6). Variables and estimates in bold show significant association (95% CI of beta excludes 0). Abbreviation: BDNF, brain-derived neurotrophic factor; BMI, body mass index

Figure 1 illustrates a significant positive association between increased log-transformed mBDNF and cognitive function, even after adjusting for covariates. Each unit increase in log-transformed mBDNF corresponds to an average rise of 1.30 standard deviations in the composite cognitive test score (*B* = 1.30, 95% CI (0.25, 2.36), *p* = 0.02). However, the initial analysis revealed issues with the assumptions of linearity, independence, homoscedasticity, and normality. A sensitivity analysis excluding influential outliers showed a consistent but smaller positive effect of mBDNF (*B* = 0.74, 95% CI (0.02, 1.47), *p* = 0.04), supporting the robustness of the findings despite initial assumption violations. Further details are available in Online Resource 1.

### Serum ProBDNF and Cognitive Function

**Fig. 2:**
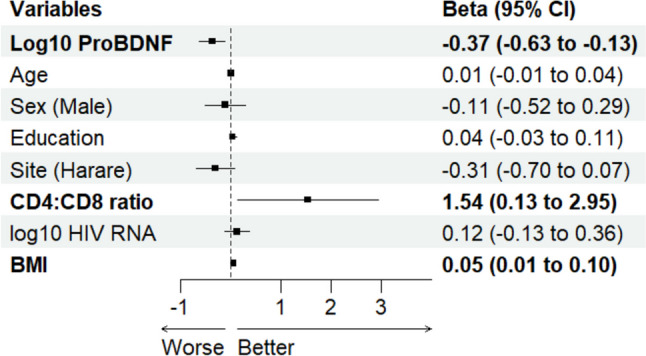
Forest plot showing the association between log-transformed proBDNF and composite cognitive score (NPZ-6). Variables and estimates in bold show significant association (95% CI of beta excludes 0). Abbreviation: BDNF, brain-derived neurotrophic factor; BMI, body mass index

Figure 2 indicates that higher levels of log-transformed proBDNF are associated with decreased cognitive performance, showing a reduction of 0.37 standard deviations in cognitive test scores for each unit increase in log-transformed proBDNF (*B* =  − 0.37, 95% CI (− 0.63, − 0.13), *p* = 0.003). However, assumptions of linearity, independence, homoscedasticity, and normality of residuals were not met. Sensitivity analysis excluding outliers showed a reduced negative effect of proBDNF on cognitive function (*B* =  − 0.15, 95% CI (− 0.33, 0.03), *p* = 0.11), though this was not statistically significant (Online Resource 2).

### Serum mBDNF/ProBDNF Ratio and Cognitive Function

**Fig. 3:**
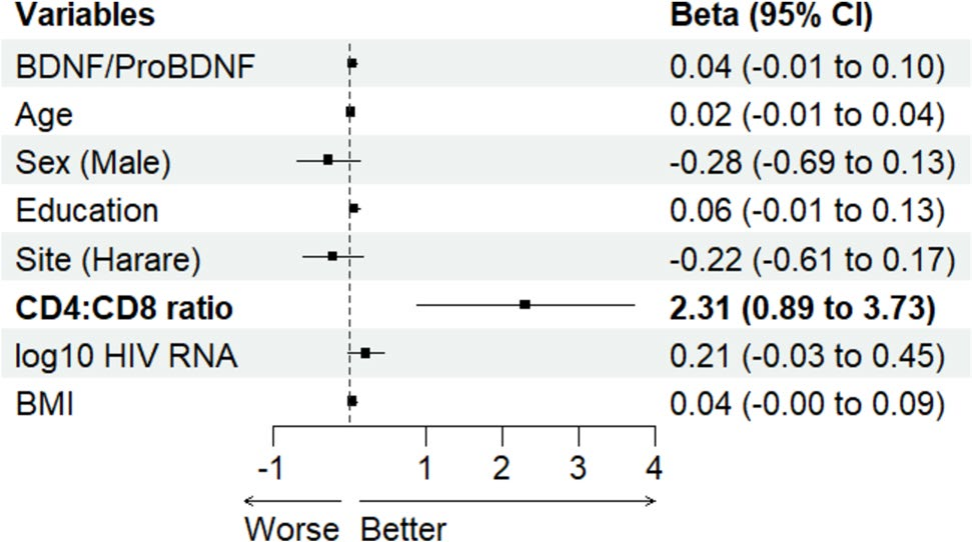
Forest plot showing the association between mature BDNF/ProBDNF ratio and composite cognitive score (NPZ-6). Variables and estimates in bold show significant association (95% CI of beta excludes 0). Abbreviation: BDNF, brain-derived neurotrophic factor; BMI, body mass index

Figure 3 shows no significant association between the mBDNF/ProBDNF ratio and cognitive performance (*B* = 0.04, 95% CI − 0.01 to 0.10, *p* = 0.08). Assumption checks indicated no major concerns with linearity, independence, or homoscedasticity. However, the Shapiro–Wilk test for normality was significant (*p* < 0.05), suggesting that residuals were not normally distributed. A sensitivity analysis excluding outliers provided a more conservative estimate (*B* =  − 0.03, 95% CI (− 0.004, 0.06), *p* = 0.09) (Online Resource 3).

### Serum BDNF Variables Across the Distribution of Cognitive Function

Table 2 shows univariate quantile regression analysis of BDNF levels on cognitive function. A significant positive association was observed for Log10 mBDNF at the median (*B* = 0.41, 95% CI (0.11, 0.72), *p* = 0.007) and 75th percentile (*B* = 1.04, 95% CI (0.01, 2.06), *p* = 0.04). Conversely, Log10 ProBDNF was significantly negatively associated at the 75th percentile (*B* =  − 0.26, 95% CI (− 0.47, − 0.06), *p* = 0.01). The mBDNF/ProBDNF ratio showed no significant association across the distribution. These univariate models were necessitated by non-positive degrees of freedom encountered in multivariable quantile regression, likely due to model overfitting or insufficient sample size at specific quantiles.

**Table 2:** Quantile regression analysis of serum mBDNF and ProBDNF variables on cognitive function

BDNF variables	Univariate quantile regression (*β* (95% CI))		
	25th percentile	50th percentile	75th percentile
Log10 mBDNF	1.00 (− 0.51, 2.51)	0.41 (0.11, 0.72)*	1.04 (0.01, 2.06)*
Log10 ProBDNF	0.17 (− 0.5, 0.17)	− 0.16 (− 0.39, 0.07)	− 0.26 (− 0.47, − 0.06)*
mBDNF/ProBDNF	0 (− 0.07, 0.07)	0.01 (− 0.04, 0.06)	0.03 (− 0.01, 0.07)

Abbreviation: *BDNF* brain-derived neurotrophic factor, *mBDNF* mature brain-derived neurotrophic factor

^*^p < 0.05

### Serum BDNF Variables and Individual Cognitive Test Scores

**Fig. 4:**
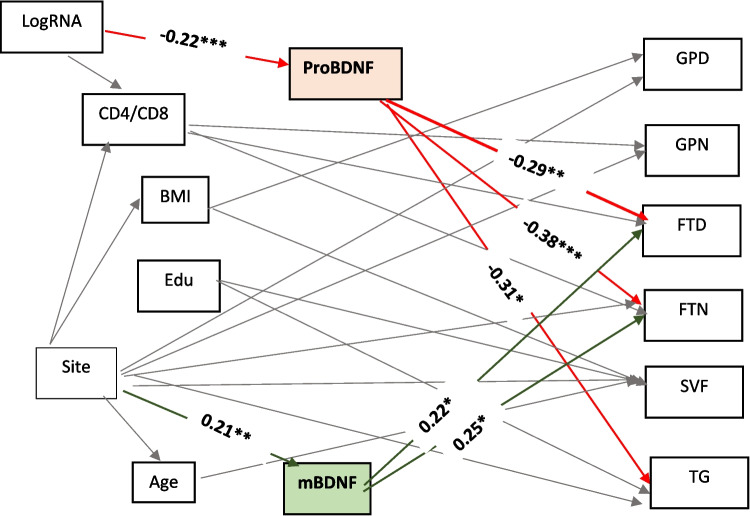
SEM path diagram of hypothesized model (*n* = 157) examining the relationships between serum mature BDNF, proBDNF, and individual neurocognitive tests. Solid lines represent significant paths (*p* < 0.05). Significant path coefficients related to mBDNF and proBDNF are bolded. Paths to and from mBDNF are highlighted in green, while paths to and from proBDNF are highlighted in red. Β-estimates shown are just for mBDNF- and proBDNF-related paths. Abbreviations: Edu, education; BDNF, serum brain-derived neurotrophic factor; GPD, Grooved Pegboard–dominant hand; GPN, Grooved Pegboard–non-dominant hand; FTD, Finger Tapping–dominant hand; FTN, Finger Tapping–non-dominant hand; SVF, semantic verbal fluency; TG, Timed Gait test. *p < 0.05, **p < 0.01, ***p < 0.001

In the SEM path diagram (Fig. 4), mBDNF was associated positively with Finger Tapping test scores for both dominant and non-dominant hands (*β* = 0.22, *p* = 0.04; *β* = 0.25, *p* = 0.03, respectively). ProBDNF showed negative associations with Timed Gait (*β* =  − 0.31, *p* = 0.02), Finger Tapping–dominant hand (*β* =  − 0.29, *p* = 0.01), and non-dominant hand (*β* =  − 0.38, *p* = 0.001). Plasma HIV RNA was negatively associated with Log10 ProBDNF (*β* =  − 0.22, *p* = 0.001), while the study site had a positive association with Log10 mBDNF (*β* = 0.13, *p* = 0.001). No significant correlation was found between mBDNF and ProBDNF (*β* =  − 0.08, *p* = 0.21). ProBDNF partially mediated the relationship between log RNA and Finger Tapping for both dominant and non-dominant hands (*β* = 0.06, *p* = 0.046; *β* = 0.08, *p* = 0.02). The model demonstrated an excellent fit (chi-square *p* = 0.37, CFI = 0.996, TLI = 0.967, RMSEA = 0.037, SRMR = 0.023). Full SEM results are available in Online Resource 4.

### Covariates

In combining results from linear regression and SEM analyses, the CD4:CD8 ratio is identified as a key predictor of cognitive function (*B* = 2.31, 95% CI (0.89, 3.73) in regression analysis; positive associations in SEM). The study site showed a negative relationship with cognitive scores in the SEM analysis (*B* =  − 0.846, *p* = 0.039 and *B* =  − 1.007, *p* = 0.002 for Grooved Pegboard dominant and non-dominant hands, respectively)), though this was not consistently significant in the linear models. Education and BMI show variable significance, indicating a complex relationship with cognitive health. Age, sex, and HIV RNA levels generally lack a significant relationship with cognitive outcomes in this cohort.

## Discussion

In this cross-sectional study, we examined the relationship between serum mature BDNF, its precursor proBDNF, and neurocognitive function in ART-naïve adults living with HIV in sub-Saharan Africa. Consistent with our hypotheses, our results reveal a significant positive association between higher serum mBDNF levels and enhanced cognitive performance. In contrast, increased proBDNF levels were linked to diminished cognitive outcomes, particularly affecting fine motor dexterity and gait speed, underscoring its potential adverse impact on neurocognition. Additionally, our analysis revealed the differential effects of these neurotrophic factors across various cognitive performance levels, with mBDNF’s influence most significant at higher cognitive percentiles. The CD4:CD8 ratio also emerged as a significant predictor of cognitive function, emphasizing the interplay between immune status and cognitive health. These insights elucidate the distinct roles of neurotrophic factors in HIV-associated cognitive impairment and underscore the need to consider both immune and neurotrophic influences in HIV neuropathogenesis. This study represents an important contribution to understanding the individual effects of serum mBDNF and proBDNF on cognitive function in ART-naïve adults with HIV.

The observed positive association between mBDNF and cognitive performance in our study aligns with existing neuroHIV research, emphasizing the critical role of mBDNF in neurogenesis and synaptic plasticity [7, 18–20, 28]. This finding echoes the results of Abassi et al., reporting an 82% reduction in the odds of cognitive impairment associated with elevated cerebrospinal fluid BDNF levels in a similar cohort of ART-naïve HIV-positive adults with advanced immunosuppression in Uganda [18]. Parallel observations have been made in ART-treated adults in developed countries, reinforcing the beneficial link between serum BDNF levels and cognitive function [20, 28]. While debates continue regarding the correlation between peripheral and central nervous system BDNF levels, evidence suggests that circulating BDNF can traverse the blood–brain barrier via a rapid saturable transport mechanism, potentially influencing brain function [29, 30].

Conversely, our study found elevated levels of proBDNF associated with diminished cognitive performance, aligning with its known role in apoptosis and synaptic pruning [14]. This is consistent with previous research that reported increased proBDNF levels in postmortem frontal cortex samples of individuals with HIV-associated dementia, likely resulting from a gp120-mediated reduction in furin synthesis, essential for cleaving proBDNF into its mature form [7].

However, our study’s findings on the mBDNF/ProBDNF ratio diverge from research in aged populations [31] and aged mice [32], suggesting the ratio’s impact on cognitive function may vary with age and health status, particularly in HIV. Unlike these studies, our younger cohort of adults living with HIV did not show a significant link between increased mBDNF/ProBDNF ratio and cognitive performance, highlighting the unique effects of HIV on neurotrophic factors. This discrepancy underscores the need for targeted research in people living with HIV to understand how the balance between mBDNF and proBDNF interacts and influences cognitive health, considering the specific challenges and characteristics of this group. The non-significant association of the mBDNF/ProBDNF ratio with cognitive performance could also suggest that the individual effects of these factors might be more critical to understand than their combined ratio.

The univariate quantile regression analysis in our study reveals distinct impacts of serum mBDNF and proBDNF on cognitive function across performance levels. Mature BDNF is positively associated with cognitive performance, particularly at median and higher cognitive levels (50th and 75th percentiles), indicating its pronounced benefits in individuals with better cognitive abilities. Conversely, elevated proBDNF levels are negatively associated with cognitive function at the higher percentile, suggesting a detrimental impact on those with initially higher cognitive performance. This pattern highlights the possibility that at lower cognitive performance levels, factors like immune status, neuroinflammation, and comorbidities may be more influential. The use of quantile regression was vital in uncovering these differential impacts, emphasizing its utility in exploring complex neurocognitive relationships. However, the study’s constraints on sample size and reliance on univariate models call for cautious interpretation and the need for further research with larger cohorts to confirm these findings.

The SEM analysis underscores mBDNF’s beneficial impact and proBDNF’s detrimental effect on fine motor skills and speed, as evidenced by their significant associations with Finger Tapping and Timed Gait outcomes. While BDNF’s role in specific cognitive domains among HIV populations has been previously documented, with higher levels correlating with improved Grooved Pegboard dominance and motor skills [19, 20], proBDNF’s influence has not been similarly studied in this demographic. Nevertheless, research in older populations [33] and stroke patients [34] indicates a negative correlation between peripheral proBDNF levels and motor function, aligning with our findings on proBDNF’s adverse effects.

Our analysis indicates a robust positive association between the CD4:CD8 ratio, a marker of immune activation, and cognitive performance, highlighting its potential as a biomarker for cognitive health in HIV [35]. Additionally, variations in cognitive performance linked to study site hint at underlying site-specific influences—genetic, environmental, or sociocultural—unaccounted for in our analysis [19, 24]. Notably, demographic factors like age, sex, and education did not consistently correlate with cognitive outcomes, likely due to the application of site-specific, demographically adjusted norms.

This study’s key strengths include its separate analysis of mature BDNF and proBDNF, the use of site-specific, demographically adjusted neuropsychological norms, and the application of robust statistical methods to clarify BDNF’s role in cognition. However, several limitations need to be acknowledged. The cross-sectional nature of the study restricts causal interpretations. There is a possibility of residual confounding, as the limited sample size did not allow for comprehensive adjustment for all potential confounders. The relatively low *R*-squared values suggest that other factors not included in the models significantly influence cognitive scores. The limited range of neuropsychological tests used limits the generalizability of our findings to other cognitive areas. Additionally, the absence of a seronegative control group hinders the establishment of baseline BDNF levels in this population, which is important for future research and for validating BDNF as a biomarker for HIV-related cognitive impairment. Furthermore, using ELISA for BDNF quantification may represent a methodological limitation. While widely used, ELISA has a lower sensitivity than more advanced multiplex assays such as Luminex or Olink. This reduced sensitivity could have affected the precision of BDNF measurements, potentially underestimating the associations between BDNF levels and cognitive outcomes, thus limiting the scope of our findings.

Another potential limitation is that BDNF levels are subject to diurnal rhythms. Research suggests that BDNF concentrations are higher in the morning and decrease throughout the day, following a circadian pattern [36]. Although we did not standardize blood draw times, variations in the time of sample collection could have impacted the BDNF levels observed in this study, influencing the associations with cognitive function. Future studies should account for diurnal variations by controlling for blood collection times. Lastly, the use of non-probability sampling and the study’s context as part of a larger clinical trial means the findings may not be widely generalizable, as our sample represents a relatively young and healthy subset of ART-naïve adults living with HIV in sub-Saharan Africa.

## Conclusion

In conclusion, this study demonstrates the differential impacts of mature BDNF and proBDNF on cognitive function in HIV, highlighting the neuroprotective role of mature BDNF and the potential detrimental effects of proBDNF. The intricate balance between these neurotrophic factors and their varying influence across cognitive performance levels underscores the complexity of HIV neuropathogenesis. Future research should focus on longitudinal studies to elucidate the temporal dynamics of mBDNF and proBDNF levels and their impact on cognitive changes during ART initiation, potentially offering new insights into targeted neurocognitive interventions in HIV. Additionally, exploring the mechanisms underlying the differential effects of BDNF and proBDNF across various cognitive domains could provide deeper insights into HIV neuropathogenesis. Establishing reference BDNF levels in seronegative controls would aid in validating BDNF as a biomarker for HIV-associated cognitive impairment. Research should also consider a broader range of cognitive tests and larger, more diverse samples to enhance the generalizability of findings and deepen our understanding of the complex interplay between neurotrophic factors, immune status, and cognitive health in HIV.

## Supplementary Information

Below is the link to the electronic supplementary material.

Supplementary file1 (DOCX 32 KB)

## Data Availability

The authors confirm that all data are fully available upon request from sdac.data@sdac.harvard.edu with the written agreement of Advancing Clinical Therapeutics Globally for HIV/AIDS and Other Infections (ACTG). Study specimens were obtained from the ACTG Repository (www.specimenrepository.org).
