# Supplementary material for: Serum Mature and Precursor Brain-Derived Neurotrophic Factors and Their Association with Neurocognitive Function in ART-Naïve Adults Living with HIV in Sub-Saharan Africa
Source: Mol Neurobiol. 2024 Nov 16;62(5):5442–51. doi: 10.1007/s12035-024-04599-2 (PMC11953136; doi:10.1007/s12035-024-04599-2)
Supplement: Supplementary file 1 — Supplementary file1 (DOCX 32 KB) [file 12035_2024_4599_MOESM1_ESM.docx]

**SUPPLEMENTARY MATERIAL**

**ONLINE RESOURCE 1**

**Serum mBDNF Levels and Cognitive Performance (Excluding Outliers)**

| **Variable** | **Estimate** | **p-value** |
| --- | --- | --- |
| Log10 BDNF | 0.74 (0.02, 1.47) | 0.04 |
| Age | 0.01 (-0.01, 0.03) | 0.24 |
| Sex (Male) | 0.01 (-0.27, 0.29) | 0.95 |
| Education | 0.03 (-0.01, 0.08) | 0.15 |
| Site (Harare) | -0.03 (-0.3, 0.24) | 0.81 |
| CD4:CD8 ratio | 1.22 (0.19, 2.26) | 0.02 |
| Log HIV RNA | 0.18 (0.01, 0.35) | 0.04 |
| BMI | 0.02 (-0.01, 0.05) | 0.16 |

Abbreviation: mBDNF, Mature Brain-derived Neurotrophic factor; BMI, Body Mass Index;

Linear regression model showing Beta estimates (95% CI)

Variables and estimates in red show significant association (95% CI of Beta excludes 0).

**ONLINE RESOURCE 2**

**Serum ProBDNF Levels and Cognitive Performance (Excluding outliers)**

| **Variable** | **Estimate** | **p-value** |
| --- | --- | --- |
| Log10 ProBDNF | -0.15 (-0.3, 0.03) | 0.11 |
| Age | 0.01 (-0.01, 0.02) | 0.42 |
| Sex (Male) | 0.10 (-0.19, 0.39) | 0.5 |
| Education | 0.04 (-0.01, 0.09) | 0.12 |
| Site (Harare) | 0.01 (-0.26, 0.28) | 0.93 |
| CD4:CD8 ratio | 1.15 (0.08, 2.22) | 0.04 |
| Log HIV RNA | 0.18 (0.01, 0.35) | 0.04 |
| BMI | 0.03 (-0.001, 0.06) | 0.06 |

Abbreviation: proBDNF, Precursor Brain-derived Neurotrophic factor; BMI, Body Mass Index;

Linear regression model showing Beta estimates (95% CI)

Variables and estimates in red show significant association (95% CI of Beta excludes 0).

**ONLINE RESOURCE 3**

**Serum mBDNF/ProBDNF Levels and Cognitive Performance (Excluding outliers)**

| **Variable** | **Estimate** | **p-value** |
| --- | --- | --- |
| BDNF/ProBDNF | 0.03 (-0.004, 0.06) | 0.09 |
| Age | -0.01 (0.01, 0.01) | 0.32 |
| Sex (Male) | 0.02 (-0.26, 0.31) | 0.88 |
| Education | -0.02 (-0.02, 0.08) | 0.2 |
| Site (Harare) | 0.02 (-0.24, 0.28) | 0.88 |
| CD4:CD8 ratio | 1.40 (0.34, 2.46) | 0.01 |
| Log HIV RNA | 0.14 (-0.04, 0.31) | 0.13 |
| BMI | 0.03 (-0.001, 0.06) | 0.06 |

Abbreviation: m BDNF, Mature Brain-derived Neurotrophic factor proBDNF, Precursor Brain-derived Neurotrophic factor; BMI, Body Mass Index;

Linear regression model showing Beta estimates (95% CI)

Variables and estimates in red show significant association (95% CI of Beta excludes 0).

**ONLINE RESOURCE 4**

**Structural Equation Modelling of Serum BDNF levels and Cognitive Test Scores**

| Estimator DWLS | | | | |  |  |  |
| --- | --- | --- | --- | --- | --- | --- | --- |
| Optimization method NLMINB | | | | |  |  |  |
| Number of model parameters 98 | | | | |  |  |  |
|  |  |  |  |  |  |  |  |
| Number of observations 157 | | | | |  |  |  |
|  |  |  |  |  |  |  |  |
| Model Test User Model: | |  |  |  |  |  |  |
| Standard Scaled | | | | | |  |  |
| Test Statistic 7.571 11.670 | | | | | |  |  |
| Degrees of freedom 7 7 | | | | | |  |  |
| P-value (Chi-square) 0.372 0.112 | | | | | |  |  |
| Scaling correction factor 0.675 | | | | | |  |  |
| Shift parameter 0.455 | | | | | |  |  |
| simple second-order correction | | | | | |  |  |
|  |  |  |  |  |  |  |  |
| Model Test Baseline Model: | | |  |  |  |  |  |
|  |  |  |  |  |  |  |  |
| Test statistic 614.041 295.309 | | | | | |  |  |
| Degrees of freedom 76 76 | | | | | |  |  |
| P-value 0.000 0.000 | | | | | |  |  |
| Scaling correction factor 2.453 | | | | | |  |  |
|  |  |  |  |  |  |  |  |
| User Model versus Baseline Model: | | |  |  |  |  |  |
|  |  |  |  |  |  |  |  |
| Comparative Fit Index (CFI) 0.999 0.979 | | | | | |  |  |
| Tucker-Lewis Index (TLI) 0.988 0.769 | | | | | |  |  |
|  | | | | | |  |  |
| Robust Comparative Fit Index (CFI) 0.994 | | | | | |  |  |
| Robust Tucker-Lewis Index (TLI) 0.936 | | | | | |  |  |
|  |  |  |  |  |  |  |  |
| Root Mean Square Error of Approximation: | | | |  |  |  |  |
|  |  |  |  |  |  |  |  |
| RMSEA 0.023 0.065 | | | | | |  |  |
| 90 Percent confidence interval - lower 0.000 0.000 | | | | | |  |  |
| 90 Percent confidence interval - upper 0.103 0.129 | | | | | |  |  |
| P-value H_0: RMSEA <= 0.050 0.617 0.299 | | | | | |  |  |
| P-value H_0: RMSEA >= 0.080 0.154 0.408 | | | | | |  |  |
|  | | | | | |  |  |
| Robust RMSEA 0.054 | | | | | |  |  |
| 90 Percent confidence interval - lower 0.000 | | | | | |  |  |
| 90 Percent confidence interval - upper 0.106 | | | | | |  |  |
| P-value H_0: Robust RMSEA <= 0.050 0.395 | | | | | |  |  |
| P-value H_0: Robust RMSEA >= 0.080 0.237 | | | | | |  |  |
|  |  |  |  |  |  |  |  |
| Standardized Root Mean Square Residual: | | | |  |  |  |  |
|  |  |  |  |  |  |  |  |
| SRMR 0.021 0.021 | | | | | |  |  |
|  |  |  |  |  |  |  |  |
| Parameter Estimates: | |  |  |  |  |  |  |
|  |  |  |  |  |  |  |  |
| Standard errors Robust.sem | | | | |  |  |  |
| Information Expected | | | | |  |  |  |
| Information saturated (h1) model Unstructured | | | | |  |  |  |
|  |  |  |  |  |  |  |  |
| Regressions: | |  |  |  |  |  |  |
| Estimate Std.Err z-value P(>\|z\|) ci.lower ci.upper Std.lv Std.all | | | | | | | |
| TimedGait ~ | | | | | | | |
| lg10BDNF1 0.016 0.144 0.110 0.912 -0.266 0.298 0.016 0.009 | | | | | | | |
| l10PBDNF1 (a3) -0.309 0.136 -2.269 0.023 -0.577 -0.042 -0.309 -0.183 | | | | | | | |
| Baseln_g1 0.105 0.131 0.804 0.422 -0.151 0.361 0.105 0.062 | | | | | | | |
| Edu_yrs1 -0.340 0.139 -2.438 0.015 -0.613 -0.067 -0.340 -0.201 | | | | | | | |
| BMI 0.046 0.128 0.358 0.720 -0.205 0.296 0.046 0.027 | | | | | | | |
| logRNA1 0.248 0.213 1.165 0.244 -0.169 0.666 0.248 0.147 | | | | | | | |
| CD4_CD8_1 0.186 0.136 1.367 0.172 -0.081 0.453 0.186 0.109 | | | | | | | |
| Site1 0.604 0.267 2.262 0.024 0.081 1.127 0.604 0.179 | | | | | | | |
| Zscore_GPD ~ | | | | | | | |
| lg10BDNF1 0.209 0.370 0.564 0.573 -0.516 0.934 0.209 0.086 | | | | | | | |
| l10PBDNF1 -0.297 0.285 -1.040 0.298 -0.856 0.262 -0.297 -0.123 | | | | | | | |
| Baseln_g1 0.063 0.105 0.598 0.550 -0.143 0.270 0.063 0.026 | | | | | | | |
| Edu_yrs1 0.104 0.132 0.790 0.430 -0.154 0.362 0.104 0.043 | | | | | | | |
| BMI 0.475 0.196 2.418 0.016 0.090 0.860 0.475 0.196 | | | | | | | |
| logRNA1 -0.061 0.179 -0.337 0.736 -0.412 0.291 -0.061 -0.025 | | | | | | | |
| CD4_CD8_1 0.235 0.147 1.599 0.110 -0.053 0.524 0.235 0.097 | | | | | | | |
| Site1 -0.846 0.411 -2.061 0.039 -1.651 -0.041 -0.846 -0.176 | | | | | | | |
| Zscore_GPN ~ | | | | | | | |
| lg10BDNF1 0.149 0.265 0.562 0.574 -0.370 0.668 0.149 0.067 | | | | | | | |
| l10PBDNF1 -0.326 0.231 -1.409 0.159 -0.779 0.127 -0.326 -0.149 | | | | | | | |
| Baseln_g1 0.094 0.149 0.633 0.527 -0.197 0.385 0.094 0.043 | | | | | | | |
| Edu_yrs1 0.216 0.164 1.317 0.188 -0.105 0.537 0.216 0.099 | | | | | | | |
| BMI 0.345 0.178 1.941 0.052 -0.003 0.694 0.345 0.157 | | | | | | | |
| logRNA1 0.009 0.176 0.053 0.958 -0.335 0.354 0.009 0.004 | | | | | | | |
| CD4_CD8_1 0.364 0.148 2.460 0.014 0.074 0.654 0.364 0.166 | | | | | | | |
| Site1 -1.007 0.326 -3.090 0.002 -1.645 -0.368 -1.007 -0.231 | | | | | | | |
| Zscore_SVF ~ | | | | | | | |
| lg10BDNF1 0.160 0.111 1.439 0.150 -0.058 0.377 0.160 0.072 | | | | | | | |
| l10PBDNF1 -0.040 0.179 -0.223 0.824 -0.390 0.310 -0.040 -0.018 | | | | | | | |
| Baseln_g1 0.422 0.146 2.896 0.004 0.136 0.708 0.422 0.193 | | | | | | | |
| Edu_yrs1 0.525 0.142 3.687 0.000 0.246 0.803 0.525 0.241 | | | | | | | |
| BMI 0.269 0.135 1.992 0.046 0.004 0.534 0.269 0.122 | | | | | | | |
| logRNA1 0.117 0.189 0.620 0.535 -0.254 0.489 0.117 0.054 | | | | | | | |
| CD4_CD8_1 0.052 0.144 0.359 0.720 -0.230 0.334 0.052 0.024 | | | | | | | |
| Site1 -3.165 0.280 -11.294 0.000 -3.715 -2.616 -3.165 -0.728 | | | | | | | |
| Zscore_FTD ~ | | | | | | | |
| lg10BDNF1 (c1) 0.202 0.112 1.803 0.071 -0.018 0.421 0.202 0.139 | | | | | | | |
| l10PBDNF1 (a1) -0.284 0.111 -2.561 0.010 -0.501 -0.067 -0.284 -0.198 | | | | | | | |
| Baseln_g1 -0.073 0.108 -0.675 0.500 -0.285 0.139 -0.073 -0.051 | | | | | | | |
| Edu_yrs1 0.132 0.100 1.327 0.184 -0.063 0.328 0.132 0.092 | | | | | | | |
| BMI 0.207 0.124 1.670 0.095 -0.036 0.451 0.207 0.143 | | | | | | | |
| logRNA1 0.112 0.112 0.997 0.319 -0.108 0.332 0.112 0.078 | | | | | | | |
| CD4_CD8_1 0.222 0.100 2.235 0.025 0.027 0.417 0.222 0.154 | | | | | | | |
| Site1 1.165 0.244 4.771 0.000 0.687 1.644 1.165 0.407 | | | | | | | |
| Zscore_FTN ~ | | | | | | | |
| lg10BDNF1 (c2) 0.239 0.128 1.862 0.063 -0.012 0.490 0.239 0.147 | | | | | | | |
| l10PBDNF1 (a2) -0.371 0.116 -3.202 0.001 -0.599 -0.144 -0.371 -0.232 | | | | | | | |
| Baseln_g1 -0.122 0.109 -1.125 0.261 -0.335 0.091 -0.122 -0.076 | | | | | | | |
| Edu_yrs1 0.085 0.118 0.715 0.475 -0.147 0.317 0.085 0.053 | | | | | | | |
| BMI 0.101 0.127 0.799 0.424 -0.147 0.350 0.101 0.063 | | | | | | | |
| logRNA1 0.173 0.130 1.327 0.185 -0.083 0.428 0.173 0.108 | | | | | | | |
| CD4_CD8_1 0.266 0.119 2.244 0.025 0.034 0.498 0.266 0.165 | | | | | | | |
| Site1 1.146 0.270 4.250 0.000 0.618 1.674 1.146 0.359 | | | | | | | |
| Site1 ~ | | | | | | | |
| lg10BDNF1 (d1) 0.128 0.030 4.220 0.000 0.068 0.187 0.128 0.252 | | | | | | | |
| BMI -0.176 0.033 -5.311 0.000 -0.240 -0.111 -0.176 -0.347 | | | | | | | |
| CD4_CD8_1 -0.089 0.040 -2.248 0.025 -0.167 -0.011 -0.089 -0.177 | | | | | | | |
| logRNA1 ~ | | | | | | | |
| CD4_CD8_1 -0.348 0.089 -3.903 0.000 -0.523 -0.173 -0.348 -0.346 | | | | | | | |
| Site1 ~ | | | | | | | |
| Baseln_g1 0.082 0.036 2.261 0.024 0.011 0.154 0.082 0.164 | | | | | | | |
| logRNA1 ~ | | | | | | | |
| l10PBDNF1 (b1) -0.220 0.069 -3.181 0.001 -0.356 -0.084 -0.220 -0.220 | | | | | | | |
|  |  |  |  |  |  |  |  |
| Covariances: | |  |  |  |  |  |  |
| Estimate Std.Err z-value P(>\|z\|) ci.lower ci.upper Std.lv Std.all | | | | | | | |
| .Zscore_TimedGait ~~ | | | | | | | |
| .Zscore_GPD 0.801 0.352 2.275 0.023 0.111 1.492 0.801 0.230 | | | | | | | |
| .Zscore_GPN 0.889 0.325 2.732 0.006 0.251 1.527 0.889 0.288 | | | | | | | |
| .Zscore_SVF 0.045 0.173 0.261 0.794 -0.294 0.384 0.045 0.021 | | | | | | | |
| .Zscore_FTD 0.479 0.133 3.610 0.000 0.219 0.739 0.479 0.251 | | | | | | | |
| .Zscore_FTN 0.424 0.150 2.830 0.005 0.130 0.717 0.424 0.198 | | | | | | | |
| .Zscore_GPD ~~ | | | | | | | |
| .Zscore_GPN 3.815 1.925 1.981 0.048 0.041 7.588 3.815 0.852 | | | | | | | |
| .Zscore_SVF 0.424 0.221 1.915 0.055 -0.010 0.857 0.424 0.139 | | | | | | | |
| .Zscore_FTD 0.922 0.406 2.273 0.023 0.127 1.718 0.922 0.332 | | | | | | | |
| .Zscore_FTN 0.524 0.213 2.460 0.014 0.107 0.941 0.524 0.169 | | | | | | | |
| .Zscore_GPN ~~ | | | | | | | |
| .Zscore_SVF 0.262 0.226 1.157 0.247 -0.182 0.706 0.262 0.097 | | | | | | | |
| .Zscore_FTD 0.672 0.304 2.211 0.027 0.076 1.268 0.672 0.273 | | | | | | | |
| .Zscore_FTN 0.398 0.211 1.883 0.060 -0.016 0.812 0.398 0.145 | | | | | | | |
| .Zscore_SVF ~~ | | | | | | | |
| .Zscore_FTD 0.246 0.138 1.786 0.074 -0.024 0.516 0.246 0.147 | | | | | | | |
| .Zscore_FTN 0.188 0.143 1.314 0.189 -0.092 0.468 0.188 0.100 | | | | | | | |
| .Zscore_FTD ~~ | | | | | | | |
| .Zscore_FTN 1.331 0.168 7.948 0.000 1.003 1.660 1.331 0.780 | | | | | | | |
| log10BDNF1 ~~ | | | | | | | |
| log10ProBDNF1 -0.095 0.064 -1.489 0.137 -0.220 0.030 -0.095 -0.096 | | | | | | | |
| Baseline_age1 0.042 0.073 0.571 0.568 -0.101 0.185 0.042 0.042 | | | | | | | |
| Edu_yrs1 -0.059 0.058 -1.005 0.315 -0.173 0.056 -0.059 -0.059 | | | | | | | |
| BMI 0.067 0.062 1.082 0.279 -0.054 0.187 0.067 0.068 | | | | | | | |
| CD4_CD8_ratio1 0.064 0.098 0.645 0.519 -0.130 0.257 0.064 0.065 | | | | | | | |
| log10ProBDNF1 ~~ | | | | | | | |
| Baseline_age1 -0.185 0.071 -2.608 0.009 -0.325 -0.046 -0.185 -0.186 | | | | | | | |
| Edu_yrs1 -0.075 0.079 -0.959 0.338 -0.229 0.079 -0.075 -0.075 | | | | | | | |
| BMI 0.164 0.087 1.890 0.059 -0.006 0.335 0.164 0.166 | | | | | | | |
| CD4_CD8_ratio1 -0.082 0.093 -0.881 0.378 -0.265 0.101 -0.082 -0.083 | | | | | | | |
| Baseline_age1 ~~ | | | | | | | |
| Edu_yrs1 -0.267 0.085 -3.158 0.002 -0.433 -0.101 -0.267 -0.268 | | | | | | | |
| BMI 0.005 0.088 0.056 0.956 -0.167 0.177 0.005 0.005 | | | | | | | |
| CD4_CD8_ratio1 -0.054 0.080 -0.669 0.503 -0.211 0.103 -0.054 -0.054 | | | | | | | |
| Edu_yrs1 ~~ | | | | | | | |
| BMI -0.058 0.072 -0.810 0.418 -0.200 0.083 -0.058 -0.059 | | | | | | | |
| CD4_CD8_ratio1 -0.130 0.088 -1.474 0.141 -0.303 0.043 -0.130 -0.131 | | | | | | | |
| BMI ~~ | | | | | | | |
| CD4_CD8_ratio1 0.091 0.071 1.285 0.199 -0.048 0.230 0.091 0.092 | | | | | | | |
|  |  |  |  |  |  |  |  |
| Variances: |  |  |  |  |  |  |  |
| Estimate Std.Err z-value P(>\|z\|) ci.lower ci.upper Std.lv Std.all | | | | | | | |
| .Zscore_TimedGt 2.398 0.445 5.393 0.000 1.527 3.270 2.398 0.839 | | | | | | | |
| .Zscore_GPD 5.053 2.897 1.744 0.081 -0.625 10.730 5.053 0.873 | | | | | | | |
| .Zscore_GPN 3.965 1.370 2.894 0.004 1.280 6.651 3.965 0.832 | | | | | | | |
| .Zscore_SVF 1.839 0.229 8.023 0.000 1.390 2.289 1.839 0.387 | | | | | | | |
| .Zscore_FTD 1.525 0.159 9.583 0.000 1.213 1.837 1.525 0.740 | | | | | | | |
| .Zscore_FTN 1.908 0.202 9.463 0.000 1.513 2.303 1.908 0.742 | | | | | | | |
| .Site1 0.191 0.014 13.268 0.000 0.163 0.219 0.191 0.758 | | | | | | | |
| .logRNA1 0.844 0.113 7.475 0.000 0.623 1.066 0.844 0.844 | | | | | | | |
| log10BDNF1 0.977 0.229 4.267 0.000 0.528 1.425 0.977 1.000 | | | | | | | |
| log10ProBDNF1 1.002 0.119 8.413 0.000 0.769 1.236 1.002 1.000 | | | | | | | |
| Baseline_age1 0.992 0.101 9.861 0.000 0.795 1.189 0.992 1.000 | | | | | | | |
| Edu_yrs1 1.000 0.123 8.160 0.000 0.760 1.240 1.000 1.000 | | | | | | | |
| BMI 0.984 0.132 7.435 0.000 0.725 1.243 0.984 1.000 | | | | | | | |
| CD4_CD8_ratio1 0.988 0.165 5.987 0.000 0.665 1.312 0.988 1.000 | | | | | | | |
|  |  |  |  |  |  |  |  |
| R-Square: |  |  |  |  |  |  |  |
| Estimate | | |  |  |  |  |  |
| Zscore_TimedGt 0.161 | | |  |  |  |  |  |
| Zscore_GPD 0.127 | | |  |  |  |  |  |
| Zscore_GPN 0.168 | | |  |  |  |  |  |
| Zscore_SVF 0.613 | | |  |  |  |  |  |
| Zscore_FTD 0.260 | | |  |  |  |  |  |
| Zscore_FTN 0.258 | | |  |  |  |  |  |
| Site1 0.242 | | |  |  |  |  |  |
| logRNA1 0.156 | | |  |  |  |  |  |
|  |  |  |  |  |  |  |  |
| Defined Parameters: | |  |  |  |  |  |  |
| Estimate Std.Err z-value P(>\|z\|) ci.lower ci.upper Std.lv Std.all | | | | | | | |
| lgRNAIndPthFTD 0.062 0.032 1.960 0.050 0.000 0.125 0.062 0.044 | | | | | | | |
| lgRNAIndPthFTN 0.082 0.036 2.279 0.023 0.011 0.152 0.082 0.051 | | | | | | | |
| logRNAIndPthTG 0.068 0.037 1.843 0.065 -0.004 0.140 0.068 0.040 | | | | | | | |
| lgRNAIPBDNFFTD 0.030 0.017 1.782 0.075 -0.003 0.064 0.030 0.037 | | | | | | | |
| lgRNAIPBDNFFTD 0.030 0.017 1.782 0.075 -0.003 0.064 0.030 0.037 |  |  |  |  |  |  |  |
